# Resveratrol aggravated H_2_O_2_-induced the HK-2 cell damage by inhibiting AKT phosphorylation

**DOI:** 10.1371/journal.pone.0327135

**Published:** 2025-07-31

**Authors:** Shicheng Luo, Yuning Chen, Xuyi Ma, Feng Tian, Xiaozhou He

**Affiliations:** 1 Department of Urology, The Affiliated Lianyungang Municipal Oriental Hospital of Xuzhou Medical University, Lianyungang, Jiangsu Province, China; 2 Department of Urology, The Third Affiliated Hospital of Soochow University, Changzhou, Jiangsu Province, China; 3 Changzhou Center for Disease Control and Prevention, Changzhou, Jiangsu Province, China; Tabriz University of Medical Sciences, IRAN, ISLAMIC REPUBLIC OF

## Abstract

**Aims:**

Reactive oxygen species (ROS) can induce renal damage when renal ischemia-reperfusion occurs as a result of factors such as renal transplant and shock. It is well established that oxidant damage plays an important role in the mechanism of ischemia-reperfusion. Resveratrol (RSV) is known for its antioxidant properties. However, in our investigations we found that RSV could aggravate human proximal tubular cells (HK-2 cells) damage when exposed to H_2_O_2_. Consequently, our findings suggested that RSV may potentially aggravate renal damage in the context of renal ischemia-reperfusion.

**Methods:**

CCK-8 reagent kits were utilized to assess cell proliferation. Western blot analysis was employed to determine the expression level of protein about γ-H_2_ax, PTEN, AKT, P-AKT and Cleave-caspase3. Additionally, the process of apoptosis was examined using flow cytometry.

**Result:**

The CCK-8 assays results revealed a dose-dependent inhibition of cell proliferation when HK-2 cells were exposed to varying concentrations of H_2_O_2_. Furthermore, when HK-2 cells were cultured with 250uM H_2_O_2_ and different concentrations of RSV, the CCK-8 results indicated a significant reduction in HK-2 cell viability with increasing RSV dosage. Flow cytometry analysis of HK-2 cells exposed to 250uM H_2_O_2_ and 10uM RSV demonstrated that RSV exacerbated apoptosis in H_2_O_2_-induced HK-2 cells. Western blot analysis revealed that RSV inhibited the expression of P-AKT and exacerbated damage to H_2_O_2_-induced HK-2 cells. Subsequently, we selected 10uM MK for further investigation, with CCK-8 results showed a significant reduction in HK-2 cell viability when the AKT phosphorylation process was inhibited by MK. Western blot analysis indicated that the cleave-caspase-3 was further activated when the AKT phosphorylation process was inhibited by MK. Flow cytometry demonstrated a significant increase in the apoptosis of HK-2 cells when the AKT phosphorylation process was inhibited by MK. In the case of P-AKT overexpression, Western blot analysis showed a significant increase in P-AKT expression, while a significant decrease in cleave-caspase-3. Flow cytometry revealed a significant decrease in the apoptosis of HK-2 cells. Finally, Western blot analysis demonstrated a decrease in P-AKT levels along with the expression of PTEN.

**Conclusions:**

Our research substantiated that RSV could aggravate H_2_O_2_ induced HK-2 cells damage by upregulating the expression of PTEN and inhibiting AKT phosphorylation. It suggested that RSV may deteriorate renal damage in the context of renal ischemia-reperfusion injury.

## Introduction

Reactive oxygen species (ROS) play a pivotal role in numerous cellular functions, including proliferation, differentiation, apoptosis, autophagy, and senescence, depending on their concentration levels [1–4]. Hydrogen peroxide (H_2_O_2_) is a well-known ROS, resulting from cellular aerobic metabolism [1,5,6]. Several reports have consistently demonstrated that ROS induced Oxidative stress to mediate the damage of cellular structure,including lipids, proteins and DNA, rely on which the imbalance between the production of ROS and the scavenging of ROS by the cell antioxidant defense system [7,8]. Over the years, numerous experiments have employed cellular models exposed to hydrogen peroxide to investigate exogenous oxidative reactions, including studies on the antioxidant properties of resveratrol (RSV) [9–11].

Resveratrol is a kind of non-flavonoid polyphenolic compound present in many plant species such as grapes, nuts and berries [12,13]. RSV was discovered in 1940, drawing attention from the scientific community due to “French Paradox” [14]. In recent years, a number of researches has revealed that RSV exhibits a wide range of biological activities, which can be anti-inflammatory, antioxidant, anti-aging, and anti-tumor [12,15,16]. RSV demonstrates its antioxidant capacity at micromolar concentrations and neutralizes oxygen radicals through various cellular pathways. Notably, RSV can exert antioxidant effects even at concentrations as low as 0.1 µM [17–21]. Additionally, RSV displays minimal cytotoxicity on HK-2 cells at concentrations below 20 µM when exposed for 72 hours [22].

In our study of RSV, we unexpectedly discovered that resveratrol does not play a protective role, even further promotes damage to renal tubular cells in hydrogen peroxide-induced renal tubular epithelial damage.

## Methods

### Materials

HK-2 cells were sourced from National Collection of Authenticated Cells Cultures (China). RPMI1640 is purchased from the Biological Industries. Fetal bovine serum, penicillin/streptomycin and 0.25% trypsin were purchased from Gibco. The CCK-8 kit is purchased from Dojindo (Shanghai). Annexin V FITC/PI apoptosis kit and the Reactive Oxygen Species (ROS) Detection Kit purchased from Beyotime. We used antibodies against human AKT,P-AKT,β-actin,Cleave-caspase3(Cell signaling Technology), PTEN (Santacruz). Resveratrol (Selleck), MK2206(MCE) and DMSO(Sigma) were used as drugs. The protein extraction kit was purchased from Keygen Bio Tech Corp, Ltd. Virus-plasmid transfection technology was supported by Jiman Biological Company. The thermostatic culture shaker was acquired from Shanghai Yiheng Technology. The horizontal electrophoresis tank and BIO-RAD Chemidoc Xrs gel imaging system are purchased from Bole Bio Rad Sub-Cell System. CO_2_ incubator, low-speed frozen centrifuge and multifunctional enzyme marker were obtained from Thermo(USA). The High-Speed Frozen Centrifuge was purchased from HITACHI (Japan).

### Cell culture

Human proximal tubular cells were cultured in 10% RPMI1640 at 37°C with 5% CO_2_. HK-2 cells used in the experiments were taken from the third generation after retrieval from liquid nitrogen. We cultured HK-2 cells in 10% RPMI1640 at 37°C with 5% CO_2_. When HK-2 cells reached 80% confluence in the culture flask, they were treated with different concentrations of RSV for 2 hours and then cultured with 250uM H_2_O_2_ for an additional 48 hours. Control group (Con Group) was treated with 0.1% DMSO in10% RPMI at 37°C with 5% CO_2_ for 48 hours. Resveratrol was dissolved into 10mM for the experimental. The lentivirus-ShRNA transfection facilitated the overexpression of HK-2 cells with technical support from Jiman Biological Corporation

### The cell proliferation and drug toxicity

The HK-2 cells were cultured in 96-hole plates and treated the cells by the same conditions as the described above. After treatment, 10ul CCK-8solution was added to each well and incubated for 1 hour. Enzyme markers were used to detect the optical.

### Western blot

After treatment, HK-2 cells were collected and protein was extracted on ice according to the provided instructions. Sample concentration was determined using the protein curve. Objective protein bands were visualized using the western blot technique. Objective protein bands were visualized using the western blot technique. ImageJ 6.0 quantitatively analyzes the grayscale value of the target western blot band.

### The HK-2 cell apoptosis

After treated in 24-hole culture plate, we washed once with PBS and added 250ul trypsin incubated cells for 5 minutes. Subsequently, 250μl 10%RPMI 1640 was added. Cells were transferred to EP tubes and washed twice with PBS. Each tube was then filled with 500μl binding buffer working fluid, 5ul Annexin V-FITC and 10ul PI. After gentle vortex mixing, samples were left at room temperature for 5 minutes and the apoptosis rate of each sample was determined by flow cytometry.

### The HK-2 cells ROS

After the cells in the culture dish have been incubated, Prepare a 1:500 dilution of DCFH-DA dye using complete medium. Aspirate the supernatant and wash the cells three times with PBS. Add the diluted dye solution and incubate at 37°C for 30 minutes in the dark. After incubation, remove the dye solution and wash the cells three times with PBS to remove excess dye. Add trypsin to detach the cells, then neutralize with complete medium. Transfer the cell suspension to a flow cytometry tube and analyze immediately. Process the acquired data using FlowJo software to generate gated populations and overlay histograms.

### The statistical analysis

All experimental data were obtained from three independent experiments. All data are presented as mean ± standard error of the mean. Comparative analyses between two groups were performed using the student t-test, while one-way analysis of variance (ANOVA) was used for comparisons among multiple groups. A P-value less than 0.05 was statistically significant.

## Results

### The effect of hydrogen peroxide on HK-2 cell viability

**Fig 1:**
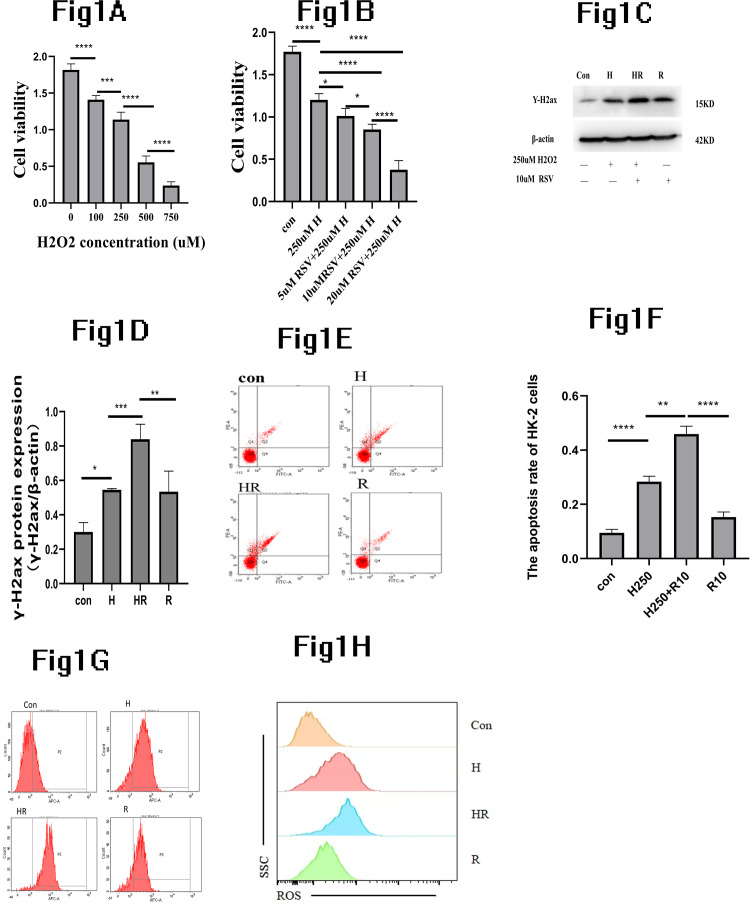
Resveratrol aggravated H_2_O_2_-induced HK-2 cell damage. (A):Detected the HK-2 cells viability cultured with the different concentrations of H2O_2_. (B):Detected the HK-2 cells viability cultured with 250uM H_2_O_2_ and different concentrations of RSV. (C): When the HK-2 cells cultured with 250uM H_2_O_2_ and 10uM RSV, western blot results showed the expression of γ-H2ax and β-actin.(D):Quantitative analysis of γ-H2ax/β-actin protein. (E&F):The flow cytometry demonstrated the apoptosis rate. (G): The flow cytometry demonstrated the ROS, (H): Convert the Ros flow chart into a fitted graph. *P  <  0.05;**P  <  0.01; ***P  <  0.001; ****P  <  0.0001.

Therefore, we selected varying concentrations of hydrogen peroxide to induce damage to HK-2 cells and tested cell viability with CCK-8 assay after 48H. We found that cell viability decreased significantly with the increase of H_2_O_2_ concentration. Cell viability between different concentrations were significantly statistically decreased. The difference is statistically significant (p < 0.5). H_2_O_2_ induced the HK-2 cells damage present dose-dependent. (Fig 1A)

### The effects of RSV on H_2_O_2_ induced- HK-2 cell damage

At the H_2_O_2_-induced HK-2 cells damage environments, we found that the HK-2 cells viability was significantly decreased with increasing RSV dose (P  <  0.05, Fig 1B). Based on the above findings, 10 µM resveratrol was selected for subsequent investigations.

### The effect of RSV on apoptosis in HK-2 cells induced by H_2_O_2_

HK-2 cells were pre-treatment with 10uM RSV for 2 hours and then deal HK-2 cells with 250uM H_2_O_2_ for 48 hours to assess HK-2 cells the expression of apoptosis. The Flow cytometry analysis revealed a significant increase in the apoptosis rate of HK-2 cells in the HR group when compared to the R group (P < 0.05, Fig 1D). The flow cytometry statistical analysis of the apoptosis rate of the HK-2 cells support this conclusion (Fig 1F).The flow cytometry analysis revealed Hydrogen peroxide significantly increased the oxidative stress, while RSV further promoted the increase of oxidative stress in HK-2 cells (Fig 1G, H).

### The γ-H_2_Ax expression of HK-2 cells with RSV treated and exposed to H_2_0_2_

we choose γ-H_2_ax to evaluate the extent of DNA damage. Our analysis revealed a substantial increase in the expression of γ-H_2_ax protein in the H group compared to the control group. More importantly, in the HR group, the expression of γ-H_2_ax protein was significantly higher than in the H group, indicating that RSV did not provide protection against H_2_O_2_-induced damage to HK-2 cells but instead exacerbated DNA damage (Fig 1C). Quantitative analysis of γ-H2ax/β-actin protein supported this conclusion (Fig 1D).

### P-AKT expression changes in HK-2 cells cultured with H_2_0_2_ and RSV

**Fig 2:**
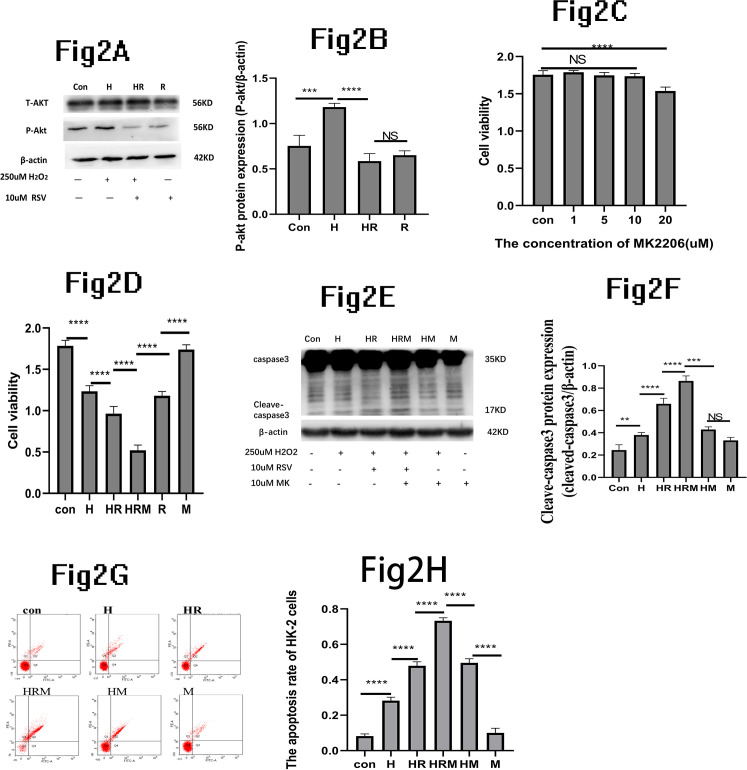
The effect of RSV in H_2_O_2_ induced damage after the AKT phosphorylation process was inhibited. (A): The western blot result showed the expressions of AKT, P-AKT and β-actin when the HK-2 cells cultured with 250uM H_2_O_2_ and 10uM RSV. (B): Quantitative analysis of AKT,P-AKT,β-actin protein. (C):Using CCK-8 detected the HK-2 cells viability cultured with MK-2206. When the AKT phosphorylation process is inhibited by MK2206. (D), Using CCK-8 detected the HK-2 cells viability. (E), The western blot showed the expressions of caspase3,cleave-caspase3and β-actin.(F), Quantitative analysis of cleave-caspase3/β-actin protein. (G), The flow cytometry demonstrated the apoptosis rate. (H), The flow cytometry statistical analysis of the apoptosis rate of the HK-2 cells *P  <  0.05;**P  <  0.01;***P  <  0.001;****P  <  0.0001;NS,There is no statistical difference.

Our findings indicated a significant increase in p-AKT protein expression in the H group compared to the control group, whereas the HR group exhibited a significant decrease in p-AKT protein expression compared to the H group. Therefore, RSV effectively inhibited P-AKT expression in H_2_O_2_-induced HK-2 cell damage (Fig 2A, B).

### Effect of MK-2206 AKT inhibitor on viability and apoptosis of HK-2 cells

The CCK-8 assays revealed that 10uM MK did not induce significant cytotoxicity in HK-2 cells. Consequently, we selected 10uM MK for further study (Fig 2C). The cell Viability of the HK-2 cells in the HRM group decreased significantly compared to the HR group, when the HK-2 cells cultured with 10uM MK (Fig 2D). On the other hand,we could find that The HK-2 cells Viability in the HR or R group increased significantly compared with that of the HRM group. Our experiment demonstrated that the expression of cleave-caspase3 increased in the H group compared to the control group, The expression of cleave-caspase3 increased in HR group compared to the H group. The expression of cleave-caspase3 increased in HRM group compared to the HR group (Fig 2E). Quantitative analysis of cleave-caspase3/β-actin protein supported this conclusion (Fig 2F).The apoptosis rate of HK-2 cells followed a similar trend with the cleave-caspase3.What’s more, the apoptosis rate of the HK-2 cells in the HRM group also increased significantly compared to the HR group (Fig 2G). The flow cytometry statistical analysis of the apoptosis rate of HK-2 cells supported this conclusion (Fig 2H). These results collectively suggest that inhibiting P-AKT leads to increase cleaved caspase-3 expression and an associated rise in the apoptosis rate.

### Induced P-AKT overexpression in the HK-2 cells

**Fig 3:**
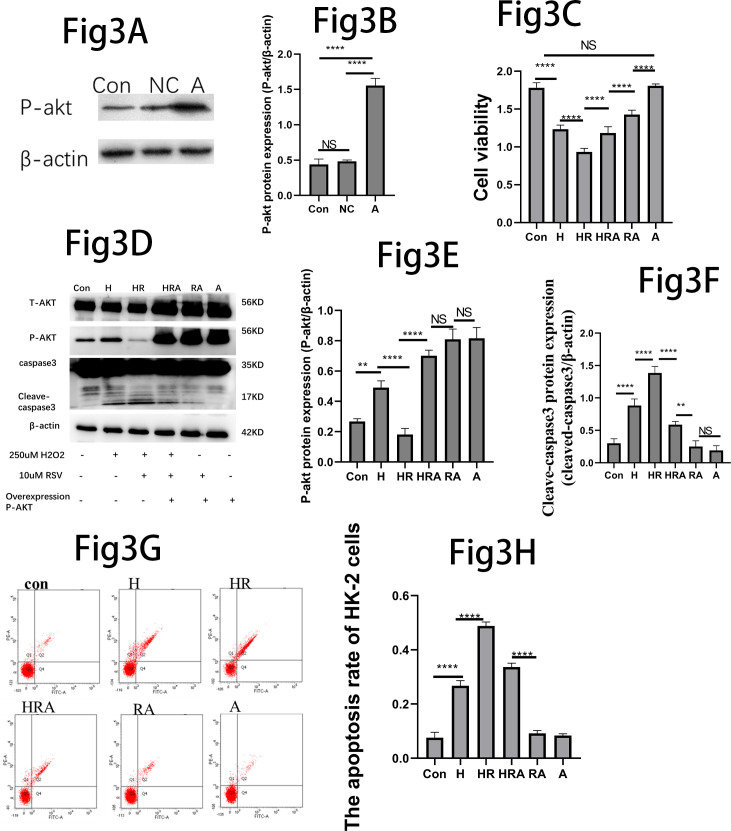
Effect of RSV in H2O2 induced damage after P- AKT was overexpressed. (A): Western blot results demonstrated the protein expression of P-AKT and β-actin. (B): Quantitative analysis of P-AKT/β-actin protein. When the P-AKT was overexpression: (C), Using CCK-8 detected the HK-2 cells viability. (D), The western blot results showed the expressions of caspase3,cleave-caspase3,P-AKT,P-AKT andβ-actin.(E&F): Quantitative analysis of P-AKT/β-actin and cleave-caspase3/β-actin protein. (G), The flow cytometry demonstrated the apoptosis rate.(H), The flow cytometry statistical analysis of the apoptosis rate of the HK-2 cells. *P  <  0.05,**P  <  0.01,***P  <  0.001,****P  <  0.0001,NS:There is no statistical difference.

We induced the P-AKT over-expression of HK-2 cells by using the lentivirus-ShRNA transfection. The transfection efficiency of over-expressed phosphorylated AKT (P-AKT) protein was verified by Western blot (Fig 3A). The CCK-8 assays revealed a significant increase in HK-2 cell viability in the HRA group compared to the HR group, whereas the HR group exhibited a notable decrease in cell viability compared to the H group (Fig 3C). On the same time, the western blot analysis showed a significant increase in P-AKT protein expression in the H group compared to the control group, the expression of P-AKT protein significantly decreased in the HR group compared to the H group; the expression of P-AKT protein significantly increased in the HRA group compared to the HR group (Fig 3D). Moreover, there was a significant increase in cleaved caspase-3 expression in the H group compared to the control group, further elevated in the HR group compared to the H group, whereas significantly decreased in the HRA group compared to the HR group (Fig 3D–F). The HK-2 cells apoptosis rate followed the same changed trend with the cleave-caspase3 (Fig 3G). The flow cytometry statistical analysis of the apoptosis rate of HK-2 cells supported this conclusion (Fig 3H).

### PTEN expression change of HK-2 cell after culture with H202 and RSV

**Fig 4:**
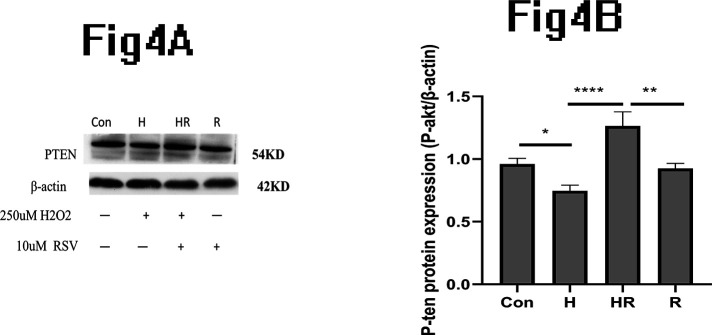
Changes in PTEN expression in HK-2 cells after cultured with H_2_0_2_ and RSV. (A): Western blot result demonstrated expression of PTEN when HK-2 cells cultured with 250uM and 10uM RSV. (B): Quantitative analysis of PTEN/β-actin protein.

We found that the expression of PTEN decreased significantly in the H group compared to the control group, while the expression of PTEN increased significantly in the HR group compared to the H group (Fig 4A). Quantitative analysis of PTEN/β-actin supported this conclusion (Fig 4B). However, the expression of P-AKT presented opposite trend compared with the expression of the PTEN (Fig 2A).

## Discussion

Renal transplantation is one of the most effective treatments for end-stage renal failure. Due to the special structure of the kidney, which makes it very sensitive to ischemia-reperfusion injury (IRI). Therefore, IRI may lead to delayed recovery of transplanted renal function, acute primary dysfunction in the transplanted kidney, acute rejection and even renal transplantation failure [23–25]. It is now understood that IRI is a complex process influenced by multiple pathways and factors, closely associated with the rapid release of oxygen free radicals, inflammation, intracellular calcium overload, apoptosis, and more [26].The explosive release of oxygen free radicals plays a pivotal role in causing cell death in renal transplantation ischemia-reperfusion injury [24].

RSV is a nonflavonoid polyphenolic compound found in many plant species including grapes, nuts and berries [12,13]. In recent years, a lot of studies had revealed that RSV has a wide range of biological activities, including anti-inflammatory, antioxidant, anti-aging, and anti-tumor [15,16,27]. However, it has also been shown to inhibit cell growth and induce apoptosis in various tumor and mammalian cell lines [28,29]. At the cellular level, RSV can induce cell death at low concentrations(≥10uM) [30–32].On the other hand, James A. Crowell confirmed that mice died when oral administration exceeds 3000 mg/kg (plasma concentration is about 6uM) RSV for more than ten days, typical of which is obvious damage to the renal tubular structure and renal function [33]. Rakesh Popat recruited 24 volunteers to participate in the experiment, taking oral RSV 5.0g per day (about 10uM in plasma). After 20 days, five of the patients developed uremia, resulting in early termination of the experiment [34]. These all suggested that RSV could cause damage to the kidney at low concentrations.

A large number of studies have focused on their beneficial or promoting tumor cell death, while few have focused on the harmful aspects of their normal cells. In our related study, a surprising phenomenon were discovered. RSV as an antioxidant in many studies and can reduce the damage caused by oxidative stress. But in our experiments, RVS did not reduce cell death in hydrogen peroxide-induced HK-2 cell damage, instead of appearing the opposite phenomenon, which aggravated the damage of HK-2 cells.

In recent years, many studies have employed hydrogen peroxide as a classical exogenous oxidative stress model for related investigations [35–37]. Therefore, we chose H_2_O_2_ to establish the cell model for research. We pre-treated HK-2 cell lines with different concentrations of RSV(5uM,10uM,20uM) after 2H exposure to the 250uM H_2_O_2_. Interestingly, we found that the HK-2 cells viability decreasing along with increasing the dose of RSV, which suggested that RSV induced the HK-2 cells death when cultured with H_2_O_2_. Accordingly, we selected 10uM RSV for further research through using HK-2 cell lines cultured with 250uM H_2_O_2_. Our study demonstrated that RSV further promoted apoptosis in the H_2_0_2_-induced damage to HK-2 cells. It has been reported that when cells are subjected to external stressors, such as radiation, drugs, or environmental factors, the DNA double-strand breaks can occur.Failure to repair such breaks promptly may lead to cell death [38]. A large number of studies had confirmed that RSV-induced DNA damage leaded to DNA double-strand exercised, which leaded to cell death [39–41]. γ-H_2_ax is a widely used biomarker to quantitatively detect the extent of DNA double-strand breaks caused by cell damage in vitro and in vivo [42–44]. Therefore, we choose γ-H_2_ax to assess the extent of DNA damage. In Our study, the expression of γ-H_2_ax significantly increased in the H group compared to the control group and this increase was more pronounced in the HR group also compared to the H group. In summary, RSV exacerbated H_2_O_2_-induced DNA damage in the HK-2 cells. RSV can promote cell apoptosis through various pathways. The apoptosis of the HK-2 cells also increased significantly in the H group compared to the control group and this increase persisted significantly in the HR group compared to the H group. To sum up, we could draw a conclusion that RSV can aggravate the damage of H_2_O_2_-induced HK-2 cells damage.

As is well known, AKT plays a pivotal role as a critical regulator of various cellular processes, including cell survival, growth, proliferation, angiogenesis, metabolism, and migration [45–47]. Resveratrol can promote cell proliferation or death by regulating AKT phosphorylation [48–50]. We observed that the expression of P-AKT protein increased significantly, when the HK-2 cells were only exposed to 250uM H_2_O_2_. However, the expression of P-AKT protein significantly decreased after 10uM RSV and 250uM H_2_O_2_. That’s mean, The HK-2 cells could be against the harmful effects of H_2_O_2_ by up-regulating phosphorylated AKT. As shown above, the expression of P-AKT protein increased significantly. Subsequently, the expression of P-AKT protein decreased significantly when the HK-2 cells were cultured with 10uM RSV and 250uM H_2_O_2_.Thus it could be seen that RSV inhibits P-AKT expression. MK-2206 is a potent allosteric AKT inhibitor, primarily blocking AKT1 and AKT2, slightly less potent against AKT3 [51]. We selected MK-2206 for further study, the HK-2 cells viability of the HRM group decreased significantly compared to the HR group, which indicated that inhibiting P-AKT led to this decrease. On the same time, the expression of cleave-caspase3 protein increased significantly in the HRM compared to the HR group. What’s more, the HK-2 cells apoptosis rate also represented the same changed trend with the cleave-caspase3 protein. Hence, we could speculate that the expression of cleave-capsase3 protein was further activated when the phosphorylation process of AKT is further inhibited. After the cleave-capsase3 protein is further activated, the apoptosis rate of the HK-2 cells also increased significantly. To validate this observation, we proceeded to overexpress P-AKT. Through lentivirus-ShRNA transfection, we achieved P-AKT overexpression in HK-2 cell lines to explore the role of RSV in regulating P-AKT in H_2_O_2_-induced damage to HK-2 cells. when the P-AKT was overexpressed, we noted a decrease in the expression of cleaved caspase-3 protein in the HRA group compared to the HR group. The apoptosis rate of HK-2 cells is decreased significantly in the HRA group compared to the HR group. what’s mean that overexpression of p-AKT can mitigate RSV-aggravated cell damage in H_2_O_2_-induced HK-2 cell damage. In summary, RSV can aggravate H_2_O_2_-induced HK-2 cells damage by inhibiting AKT phosphorylation.

PTEN acts as a regulator of PI3K and through dephosphorylating PIP3 into PIP2, inhibits AKT phosphorylation, thus regulating cell growth and proliferation [52,53]. Accord to it, we speculate that the PTEN maybe play a role in this study. Finally, The expression of P-AKT exhibited an opposite trend compared to the PTEN. Therefore, it can be inferred that PTEN inhibits AKT phosphorylation.

## Conclusion

In summary, our research substantiated that RSV can aggravate H_2_O_2_-induced HK-2 cells damage by upregulating the expression of PTEN and inhibiting AKT phosphorylation. It suggested that RSV may deteriorate renal damage in the context of renal ischemia reperfusion injury.

## Supporting information

S1 File Imformation. (RAR)
